# Gut microbiome responses to dietary intervention with hypocholesterolemic vegetable oils

**DOI:** 10.1038/s41522-022-00287-y

**Published:** 2022-04-11

**Authors:** Rachel Rui Xia Lim, Mi Ae Park, Long Hui Wong, Sumanto Haldar, Kevin Junliang Lim, Niranjan Nagarajan, Christiani Jeyakumar Henry, Yuan Rong Jiang, Oleg Vladimirovich Moskvin

**Affiliations:** 1 WIL@NUS Corporate Laboratory, National University of Singapore, Centre for Translational Medicine, Singapore, 117599 Singapore; 2 Clinical Nutrition Research Centre (CNRC), Singapore Institute for Clinical Sciences (SICS), Agency for Science Technology and Research (A*STAR), Singapore, 117609 Singapore; 3 Computational and Systems Biology, Genome Institute of Singapore (GIS), Singapore, 138672 Singapore; 4 Wilmar (Shanghai) Biotechnology R & D Center Co., Ltd., 200137 Shanghai, China; 5 Present Address: Division of Chemistry & Biological Chemistry, School of Physical and Mathematical Sciences, Nanyang Technological University, Singapore, 637371 Singapore

**Keywords:** Microbiome, Metagenomics, Health care, Metagenomics

## Abstract

Hypercholesterolemia is becoming a problem with increasing significance. Dietary vegetable oils may help to improve this condition due to presence of phytonutrients with potentially synergistic cholesterol-lowering effects. The objective of this 8-week double-blinded randomized clinical trial was to investigate the effects of consuming 30 g of two different blended cooking oils, rich in omega-3 alpha-linolenic acid and phytonutrients, or refined olive oil on the intestinal microbiota in 126 volunteers with borderline hypercholesterolemia. Multi-factor analysis of relationships between the gut microbiota composition at various taxonomic ranks and the clinical trial parameters revealed the association between beneficial effects of the dietary intervention on the blood lipid profile with abundance of *Clostridia* class of the gut microbiota. This microbiota feature was upregulated in the course of the dietary intervention and associated with various plasma markers of metabolic health status, such as Triglycerides, Apolipoprotein B and Total Cholesterol to HDL ratio in a beneficial way. The relative abundance of a single species—*Clostridium leptum*—highly increased during the dietary intervention in all the three study groups. The oil blend with the highest concentration of omega-3 PUFA is associated with faster and more robust responses of the intestinal microbiota, including elevation of alpha-diversity. Butyrate production is being discussed as a plausible process mediating the observed beneficial influence on the plasma lipid profile. Causal mediation analysis suggested that *Clostridium* genus rather than the higher rank of the phylogeny—*Clostridia* class—may be involved in the diet-induced improvements of the blood lipid profile.

## Introduction

It is becoming increasingly clear that the enormous metabolic potential of human gut microbiome shaped up by early life, environment and the diet^1^ is responsible for a large part of the interaction between the diet and the human health^2,3^. Microbiota-dependent diet modulation affects autoimmunity^4^, hypertension^5^, obesity and diabetes^6^. Wider appreciation of the Diet–Microbiome–Health axis resulted in the “Food Pharmacy” term recently coined^7^ and “food as medicine” concept being actively promoted^8^, with increasing attention to individualization of the “food therapies” based on the individual microbiome signatures^9^.

The initiatives like Integrative Human Microbiome Project^10^, while not being explicitly diet-focused, are helping to understand general patterns of microbiota-health interaction at a mechanistic level, and a number of clinical trials aimed to investigate the role of this interface in particular health conditions are being conducted across the globe^11^.

Still, the range of health phenotypes addressed by the HMP is limited, and specialized studies are needed to address the role of gut microbiome in conditions such as hyperlipidemia, in context of the geographically-distinct populations that may have specific gut microbiome composition. One of the main food components widely associated with both the improved function of gut microbiome and host health benefits is dietary fiber demonstrated to increase the Prevotella/Bacteroides ratio and microbial diversity in the gut and facilitate production of health-promoting short-chain fatty acids^12^. Recently, influence of dietary oils is also gaining attention. A recent meta-analysis of 17 dietary intervention clinical trials targeting hypercholesterolemic subjects^13^ revealed that plasma triglycerides may be reduced with omega-3 fatty acids dietary supplementation. The latter also showed a trend to reduce total and LDL cholesterol, underlying the importance of the omega-3 polyunsaturated fatty acids (PUFA) for decreasing the risk of cardiovascular diseases extensively explored earlier^14^. The effects of the dietary lipids on host phenotype have been also shown to be substantially mediated by the gut microbiota^15^; this phenomenon belongs to a more general interplay between the microbiota and the human metabolism^16^.

In a recent mouse study, a reversal of detrimental effects associated with high-fat diet was shown with the supplementation of three types of dietary oils, detectable as attenuation of the decrease of *Bifidobacteria* and suppression of the increase of the *Enterobacteriaceae* in the murine gut^17^. Notably, this particular study was culture-based rather than metagenomics-based. Microbiota may affect host’s HDL metabolism^18^, and there is accumulating evidence on the gut microbiota mediation of the beneficial effects of dietary oils on inflammatory conditions via various effects (either immunostimulatory or immunosuppressive) of lipopolysaccharide isoforms produced by different microbes^19^. The anti-diabetic effects of PUFA were shown to be mediated by certain microbiota species, including those of *Ruminococcus* genus^20^.

In a recent study of dietary intervention with blended oils^21^ beneficial effects of two blended oils on blood lipid profile similar to the effect of olive oil were demonstrated. The present study, being a sub-study of that dietary intervention clinical trial is focused on assessing the responses of the intestinal microbiota of borderline hypercholesterolemic Chinese adults to an 8-week supplementation with dietary oils of various compositional signatures: refined olive oil (high in MUFA) and two novel oil blends (high in omega-3 PUFA, ALA). Comparison of the results of two lines of associations: (1) dynamics of the microbiota features following the dietary intervention and (2) correlations of the microbiota features with the blood markers of metabolic status suggested the engagement of *Clostridia* in diet-microbiota-health axis via connecting the beneficial oil consumption and improvements of the plasma metabolic marker profiles.

## Results

### Preservation of the individual microbiome signatures during the dietary intervention

**Fig. 1:**
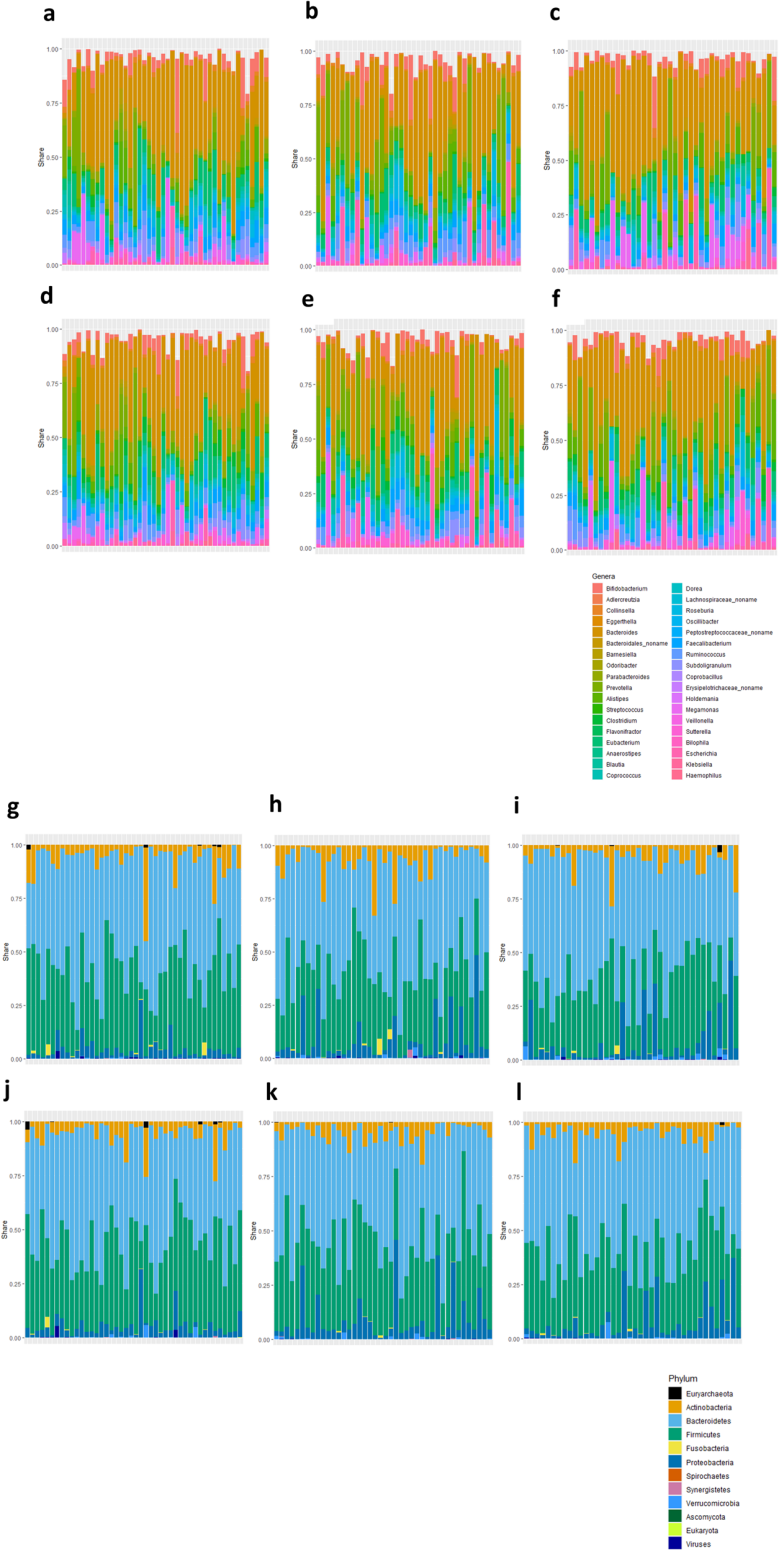
Sample-level relative abundances at different ranks of taxonomy. **a**–**f** the 36 most common bacterial genera; **g**–**l**, phyla. **a**–**c** and **g**–**i**, study groups A,B,C before the dietary intervention (Week 0); **d**–**f** and **j**–**l** study groups A,B,C at the completion of the trial (Week 8).

Figure 1 shows sample-centric shares of the most common bacterial genera (36 genera, selected by being detected in at least 40% of the samples across the dataset), as well as sample profiles at phylum level of the taxonomy. From comparison of the profiles on the top and bottom rows for each taxonomy level (i.e., data for the same study subjects before and after the clinical trial), it is evident that the degree of the variance between the individuals exceeds any changes that may be caused by the 8-week dietary intervention. We respected this observation by choosing the analytical strategy that prioritizes detection of the within-subject changes during the intervention.

More detailed profile of the microbial entities at 6 phylogeny ranks is given Suplementary Table 2. Out of 387 bacterial species ever detected in the dataset, only 1 (*Ruminococcus obeum*) was found in all the 378 samples. Median value of the share of samples with non-zero detection of a particular bacterial species is 5%. As expected, this number increases when we move up the phylogeny tree, reaching 10% at the Class level. Still, individual signatures are preserved at this phylogeny level as well, beyond the 6 classes that are detected in over 95% of the samples. At the species-level, relationship between the overall abundance in the dataset and frequency of the detection in an individual sample is not straightforward: the enterotype-associated *Prevotella copri*, while being the most abundant overall (6.4% of the pooled dataset, almost an order of magnitude more than the “housekeeping” *R.obeum* at 0.77%) was detected in 39% of the samples only, showing an “on-off” detection pattern.

**Fig. 2:**
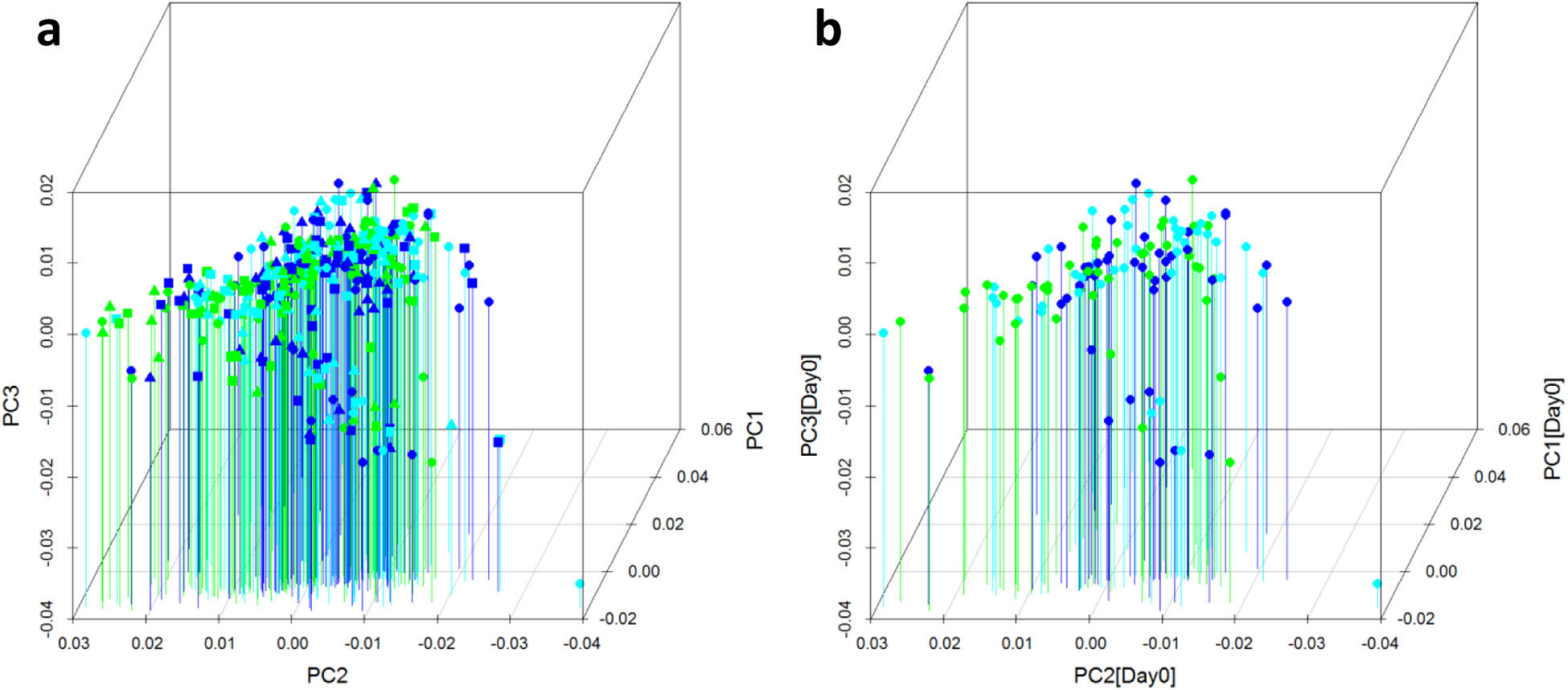
The top 3 principal components of the PCA analysis. **a** Entire set of the 378 samples (126 subjects, 3 time points); **b** Visualization is restricted to the 126 Week 0 (pre-intervention) samples. Green, study group A; blue, group B; cyan, group C. Circles, Week 0; squares, Week 2; triangles, Week 8.

A more formal analysis of relative contribution of different study factors in the metagenomic data variance (PCA, Fig. 2) shows that the landscape formed by the 3 first principal components is not significantly influenced by either Group or Time factors of the study (Fig. 2a). At the same time, restricting the visualization of the same PCA analysis to the pre-intervention time points (Week 0) shows conservation of the PCA landscape; thus, the latter is formed by the differences in the pre-existing individual microbiome signatures. PERMANOVA (Supplementary Table 4) analysis shows that indeed, 48.17% of variance (*p* = 0.0009), was explained by inter-individual variability, while the variance explained by group and time was 0.75% (*p* = 0.0009) and 0.57% (*p* = 0.0009), respectively. This phenomenon prompted us to choose a conservative approach of identification the intervention-related microbial changes that relies on the individual-centric temporal changes of the feature abundance (see Methods).

### Microbiome dynamics in the course of the dietary intervention

#### Diet-related changes in the microbial diversity

**Fig. 3:**
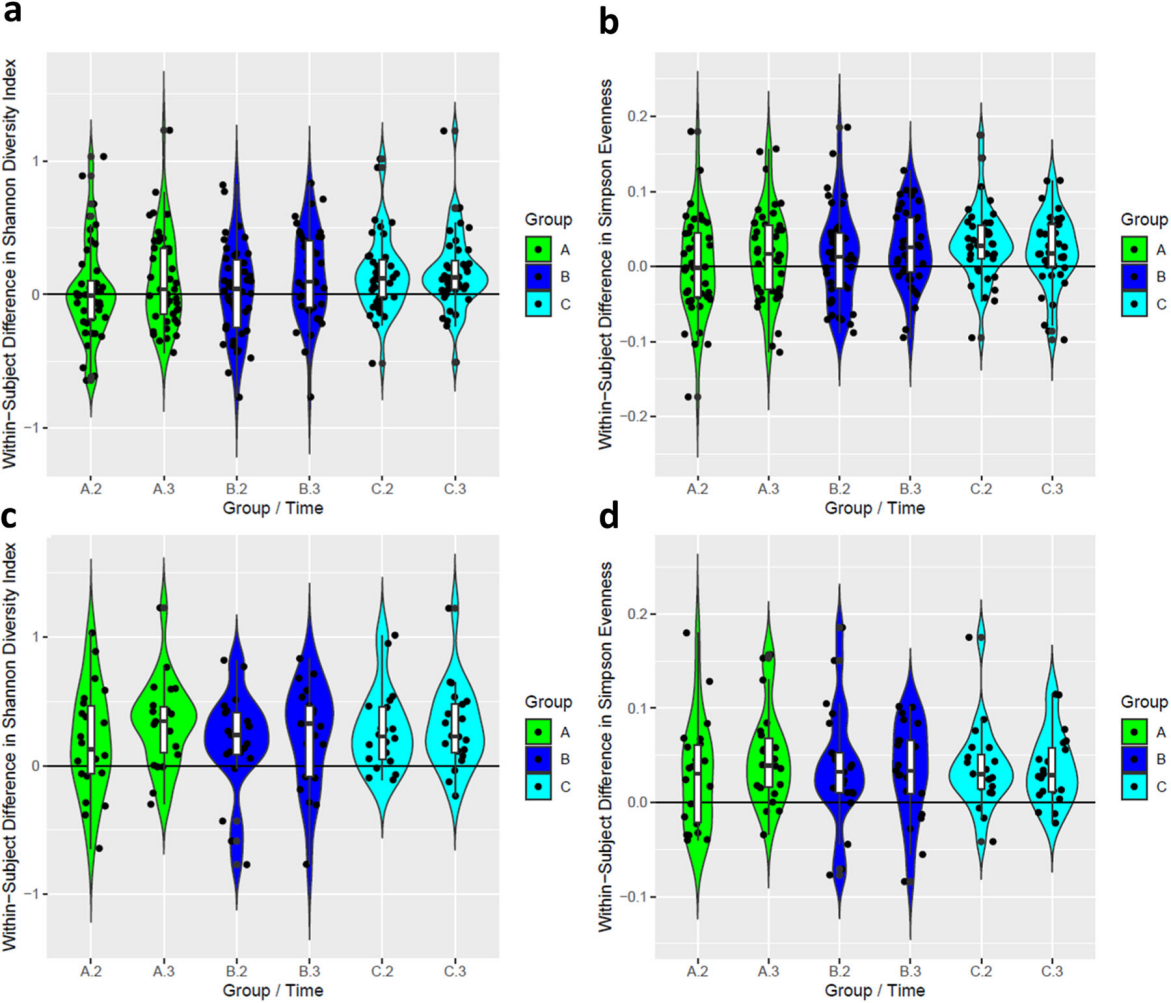
Effect of the dietary intervention on the per-subject changes in gut microbial diversity. Distributions of the differences in the diversity statistics (**a** and **c**—Shannon Diversity Index, **b** and **d**—Simpson Evenness Index) for 6 combinations of study group and the time point number. Group/Time labels: A2—per-subject differences between the second time point (Week 2) and Week 0. A3—per-subject differences between the third time point (Week 8) and Week 0, the same for study groups B and C. Panels **a** and **b,** the entire dataset. Panels **c** and **d,** dataset restricted to 50% subjects (within each group) demonstrating the lower Shannon diversity before the start of the trial. Ranges of the absolute values of the indices across the entire dataset: Simpson Evenness: 0.025 to 0.377, Shannon Diversity: 1.41 to 3.71.

One of the characteristics of the gut microbiome that is constantly being referred to as beneficial for the human health in different phenotypic contexts is microbial diversity. We computed two statistics describing the phenomenon—the Shannon Diversity Index, as well as Simpson Evenness Index, in our metagenomics dataset. By the end of the dietary intervention, the diversity indices increased in all the 3 groups (Fig. 3a, b), being statistically significant in groups B and C and borderline significant in group A (Table 1, Week 8 vs. Week 0, all data), according to the paired Wilcoxon test. Notably, if we restrict the dataset to the 50% of the subjects that demonstrated the lower Shannon diversity before the start of the trial (“bottom 50%”), subsequent increase in the diversity indices in the less responding groups (A and B) become more significant (Fig. 3c, d and Table 1 “Bottom 50%”). This suggests that the dietary intervention increased the microbial diversity preferably in the subjects that had lower initial microbial diversity.

**Table 1:** *p*-values of the paired Wilcoxon test for differences between the Shannon diversity indices and Simpson Evenness Indices, computed for the Week 2 or Week 8 samples, as compared to the diversity indices in the samples of the respective individuals collected before the intervention (Week 0).

Comparison	Statistic	Dataset version	Group A	Group B	Group C
Week 2 vs. Week 0	Shannon Diversity	All data	0.79	0.69	0.0012
		Bottom 50%	0.059	0.032	0.00039
	Simpson Evenness	All data	0.98	0.26	0.00013
		Bottom 50%	0.030	0.010	0.00048
Week 8 vs. Week 0	Shannon Diversity	All data	0.11	0.016	0.00032
		Bottom 50%	0.00015	0.011	0.00048
	Simpson Evenness	All data	0.064	0.035	0.0042
		Bottom 50%	0.000081	0.0080	0.00032

Datasets versions: “All data”, the complete sets of samples, “Bottom 50%”—subset of samples restricted to subjects that had below the group-wide median value of the Shannon diversity index (22 subjects out of 44 in Group A, 21/42 in Group B and 20/40 in Group C).

Comparing the inter-group differences of the effect on Shannon diversity (i.e., progressive rise in both the magnitude and speed-of-establishment of the elevated microbial diversity from groups A to B to C) with the differences in the compositions of the oil supplements (Supplementary Table 1), a compositional pattern that follows this trend is the concentration of the alpha-linolenic acid, ALA (0.8/20.9/34.0 percent of the total fatty acids in groups A/B/C) and the corresponding omega-3 to omega-6 PUFA ratio (0.09/0.70/1.44). Table 1 from the earlier publication^21^ shows that no other oil compounds accounted for in this study have similar distribution across the 3 oils. While we cannot exclude a combinatorial effect of some unaccounted minor components of the oil blends, the ALA concentration pattern across the three oil types stays as a possible explanation of the effect on diversity.

#### Species dynamics

**Fig. 4:**
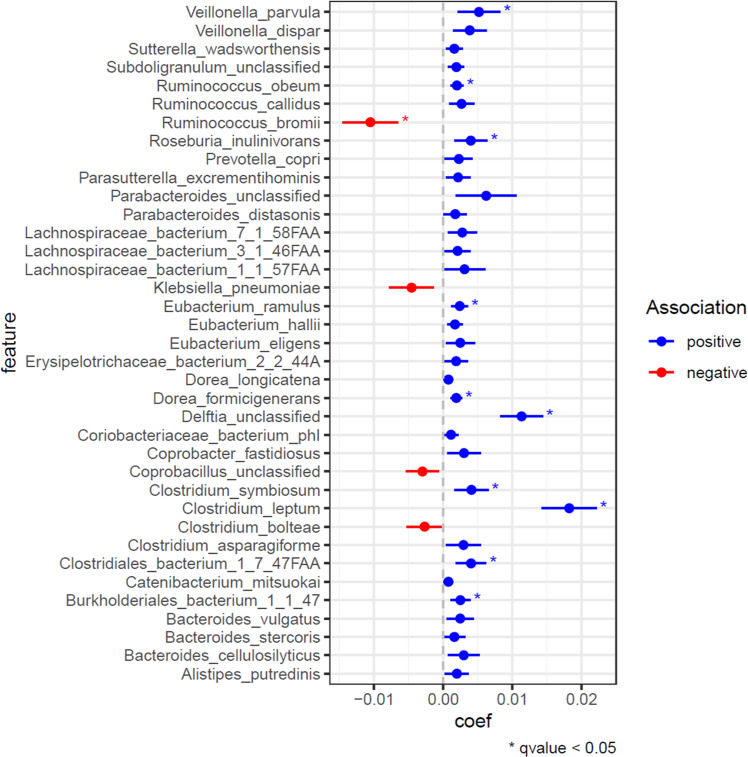
Associations of bacterial species with “Time” parameter of the *MaAsLin2* multivariate regression model. The top 37 species by the significance of the association are shown (*p* < 0.05). Associations with the *q*-values below 0.05 are marked with asterisks. Positive associations—species which abundance increased in the course of the dietary intervention; negative associations—species with decreased abundance. Error bars represent standard deviation.

In the course of the dietary intervention, 11 bacterial species changed their relative abundance (FDR < 0.05, Maaslin2), including 3 *Clostridium* species (Fig. 4). Five *Clostridium* species were among the top 37 species ranked by significance; 4 of them showed a tendency to increase in abundance in the course of the clinical trial. Within the *Ruminococcus* genus, we see species-level restructuring, with *R.obeum* and *R.callidus* increased and *R.bromii* decreased. Among the four species on Fig. 4 that show a trend to be downregulated by the intervention, the next-largest coefficient after *R. bromii* belongs to the pathogenic *Klebsiella pneumoniae*, however, without reaching the formal statistical significance. Representatives of known metabolically beneficial *Roseburia*^22^, *Vellionella*^23^ belonged to the top 11 list and showed a trend to increase their abundance, while *Prevotella*^24^ and *Sutterella*^25^ demonstrated the uptrend with lower significance. The top responsive species, *Clostridium leptum*, showed the statistical significance level 4 orders of magnitude better compared to the next responder species in the ranked list, as well as the highest coefficient of the association. No associations with the study group (with FDR < 0.05) were detected (Supplementary Table 6).

*C. leptum* could also be detected as a species increased in abundance following the dietary intervention, when running MaAsLin2 separately within the three study groups, albeit with attenuated but still statistically significant FDR values.

**Fig. 5:**
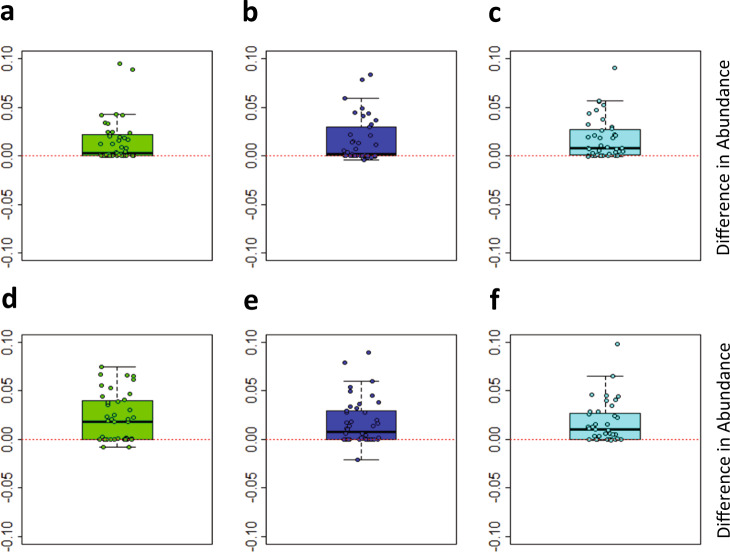
Distribution of per-subject shifts in relative abundance of *Clostridium leptum* after 2 and 8 weeks of the dietary intervention. **a**–**c** Differences between Week 2 and Week 0; **d**–**f** Differences between Week 8 and Week 0. **a**, **d**—study group A (green); **b**, **e**—study group B (blue); **c**, **f**—study group C (cyan). Borders of the box plot represent upper and lower quartiles, the bold line—median value, and the whiskers—the most distant data points that stay within 1.5 of the interquartile range from the median.

While testing for the differential abundance within each study group separately may have less power, especially in detecting common signals preserved across groups (illustrated by the just-mentioned *C.leptum* case), it is a cleaner method to detect a group-specific signals, considering the existence of a false group-related signals in the dataset driven by individual variations, that might not be entirely removed by the multivariate model. To achieve this, we used paired Wilcoxon test between the time points within each study group. This provides both group isolation and a natural way of handling the subject-specificity (paired test mode). Supplementary Table 7 shows the results of this test for the species dataset. *Clostridium leptum* was confidently detected in all the 6 comparisons (3 groups, 2 time points per group) as a species with highly increased abundance, with 6-to-31-fold change difference above the Week 0 level in different comparisons (column B) and an absolute shift in relative abundance nearly an order of magnitude larger than the corresponding value of the second strongest responder species (column C). Visualization of per-subject differences in *C.leptum* abundance across the study groups and time point comparisons is given on Fig. 5.

#### Dynamics at higher levels of phylogeny

Since higher ranks of the phylogeny are often discussed as representative for differential microbiome changes in various studies (up to the phylum-level *Firmicutes* to *Bacteriodites* ratio^6^), we further analyzed the intervention parameters associations with microbiome features at other ranks of the phylogeny via *Maaslin2* multivariate model. Similar to the case of species-centric associations, the “Group” factor was not significantly associated with any microbial feature across the higher phylogeny ranks, while the “Time” factor was (Supplementary Table 8).

The top upregulated entity at the family level was *Clostridiaceae*, with the largest coefficient and a highly significant FDR level of 2.05 × 10^−13^. While this was not the only responsible entity at this phylogeny rank according to the multivariate model, others were neither supported by an independent Wilcoxon test for the difference between the time points (Supplementary Table 7) nor showed up in blood marker association test (below). An order-level upregulation signal, *Clostridiales*, does not seem to be a consequence of the single-species *C. leptum* effect but the result of summarization of smaller upregulation effects (with lower statistical significance) for other genera within this class, such as *Dorea*: *D.unclassified, D. formicigenerans, D. longicatena* are found high on the ranked list in different explicit time point comparisons (Supplementary Table 7), *Eubacterium* (high ranks of *E. ramulus, E. limosum, E. hallii* and *E. eligens* in Supplementary Table 7), as well as other less upregulated *Clostridium* species (*C. asparagiforme, C. nexile, C. symbiosum*). This branch of the phylogeny also shows stronger statistical upregulation at a higher taxonomical level—class *Clostridia*.

Moving forward, *Clostridia* response across the levels of the taxonomy was observed in a different type of analysis— associations between taxa abundance and the blood markers of metabolic health status.

#### Associations between the microbiome composition and the blood markers of metabolic health

In the earlier publication based on the clinical data of this trial, improvements in the readings of the blood markers of the metabolic health as a result of the dietary intervention have been reported^21^. This included decrease in total and LDL cholesterol, triglycerides, apolipoprotein B, as well as the ratios of Apolipoprotein B to A1 (ApoB/ApoA1) and the total cholesterol to HDL cholesterol.

**Fig. 6:**
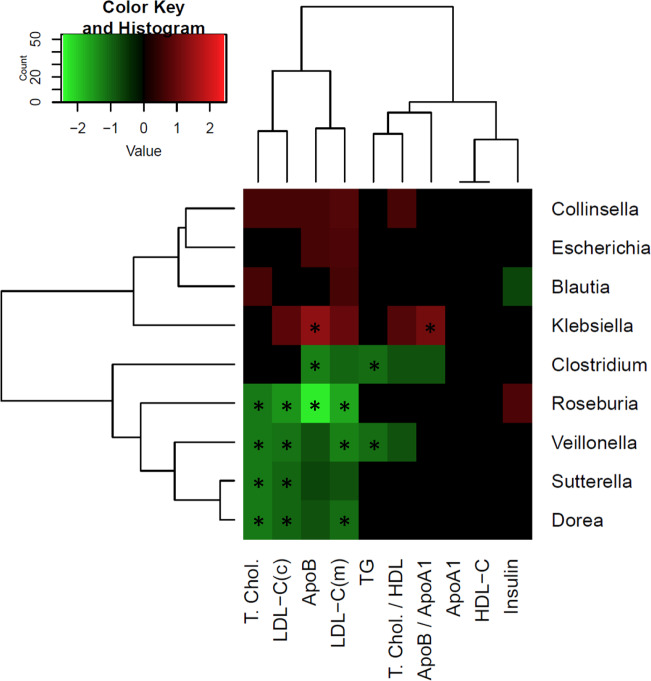
Associations of the bacterial genera abundance and the blood markers of metabolic health. The represented features passed the default *Maaslin2* significance cutoff (FDR < 0.25) in association with at least 2 out of 10 parameters: ApoA1, ApoB, ApoB/ApoA1 ratio, insulin, triglycerides (TG), total cholesterol (T. Chol), measured and computed LDL-C (LDL-C(m), LDL-C(c)), HDL-C and the Total Cholesterol to HDL ratio (T.Chol / HDL). Negative logs of the False-Discovery Rate are visualized, with values corresponding to FDR > 0.25 set to zero (black) and values multiplied by –1 for cases of negative associations. Stars, FDR < 0.1.

Based on previous reports associating gut microbiota with blood markers of metabolic health^26,27^ or lipid metabolism^28^ we hypothesized that the gut microbiota changes due to the consumption of different oils in this study may be involved in regulating the blood lipid profile. The overview of the associations between the blood marker levels and relative abundance of bacterial genera is presented on Fig. 6. Nine genera showed at least borderline associations (FDR < 0.25) with two or more of the 10 blood parameters indicated by non-black patches. The complete association data is recorded in the Supplementary Table 9. *Veillonella* genus demonstrated the highest number of the individual associations with the clinical parameters that passed the relaxed FDR cutoff (6 out of 10), with 4 associations passing the FDR < 0.1 cutoff, demonstrating consistently beneficial type of the association by being negatively associated with Total Cholesterol, LDL, ApoB, Triglycerides and Total Cholesterol to HDL ratio. Veillonella was associated with increased athletic performance^23^ due to its lactate utilizing capability. *Roseburia* genus, already noticed earlier as a beneficial phylogeny branch that increased in abundance during the dietary intervention, showed the strongest beneficial association with ApoB level. *Clostridium* genus was the second in the beneficial list by the number of favorable associations (5) with the blood markers.

In an earlier work exploring microbial associations with the blood metabolic health markers, 2 out of 3 microbial clusters that showed strong association with blood HDL levels are related to *Clostridiaceae*^26^. The study also reported negative correlations of the abundance of *Clostridiaceae* family with BMI and triglycerides levels. In our study, we see metabolically beneficial association patterns for this phylogeny branch at several levels, starting from *C.leptum* species to *Clostridium* genus to *Clostridiaceae* family to *Clostridiales* order to *Clostridia* class (Supplementary Table 9). This table also shows that when moving up the taxonomy tree, the strength of blood marker associations tends to increase.

**Fig. 7:**
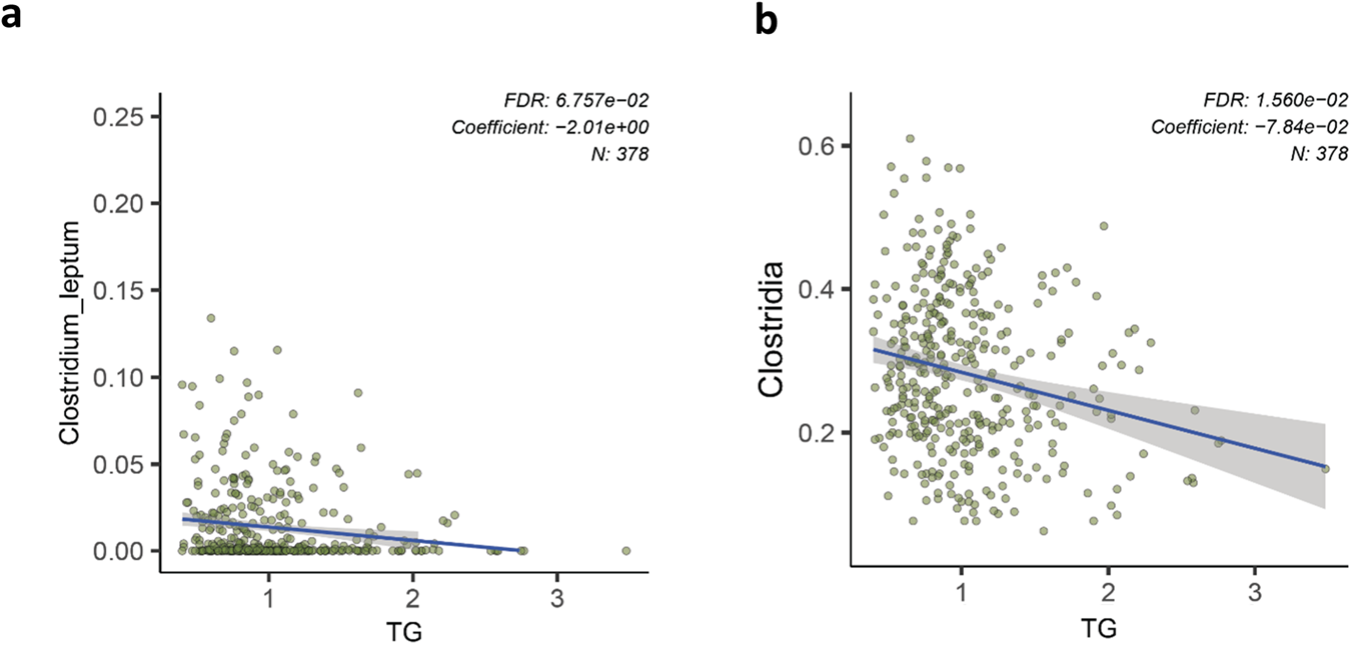
The beneficial association between *Clostridia* abundance in the gut microbiome and plasma triglyceride levels is more pronounced at higher ranks of the phylogeny. **a** Species-level association (*C. leptum*); **b** Class-level association.

Figure 7 illustrates the underlying sample-centric relationships between the abundance of entities of this taxonomy branch and the Triglycerides (TG) levels, comparing species-centric (a) and class-centric (b) results. Each scatter plot provides the basis for the summarized, single-number representation of the associations seen on Fig. 6. This suggests that the class-level beneficial metabolic health association for *Clostridia* is not an echo of a more particular phenomenon existing at the lower phylogeny rank but rather a true class-level association.

Among other order-level associations we see in Supplementary Table 9, a long metabolically disadvantageous association pattern for *Enterobacterales* is not surprising because members of this order largely contribute to dysbiotic state of the intestine typically associated with health conditions^29^. Besides *Clostridiales*, *Burkholderiales* order also show beneficial blood markers associations. *Burkholderiales* encompasses such genera as *Delftia, Oxalobacter, Parasutterella* and *Sutterella*, from the inspection of the species- and genus-level signals it looks like this signal is driven by *Sutterella*. For *Clostridia*, a combination of beneficial blood marker associations at several taxonomy ranks with increase in their relative abundance in response to the hypocholesterolemic oils—also at several taxonomic levels—represents a consistent story on the beneficial gut microbial compositional pattern. It is tempting to interpret the fact of increasing beneficial correlation strength from lower to higher phylogeny ranks of the *Clostridia* branch as an indication of the functional importance of the class-level phylogenetic entity in promoting the metabolic health of the host. However, the relation between the correlations and the functional importance is not that straightforward.

Considering the biological functionality, we expected to see a differential abundance signal related to Time (and possibly, Group) factor when the metagenomic data is represented as pathway abundance. After applying the same statistical approaches to pathway-level data, no pathways passed the FDR cutoff (Supplementary Tables 10 and 11). To avoid coming up with unreliable conclusions, we do not discuss pathway-level changes here.

To shed some light on the question if the observed phylogeny to blood markers correlations indicate any causal link between the changes in microbiota composition and the levels of blood markers, we applied causal mediation analysis^30^ to the select taxa—blood marker pairs (Table 2). In context with the previous analysis, *Clostridia*-related pairs, especially those showing the highest correlation levels (i.e., TG) were of the main interest. Table 2 shows that the proportion of the diet intervention-caused decrease in Triglyceride levels mediated by the increase in *Clostridium* genus is estimated to be around 15%, with uncertain confidence interval that goes up to 55%. Still, the p-value to call the mediation effect was not significant (0.136). While the upper confidence intervals for mediation effects in the case of *Clostridia* class and *C.leptum* species in relation to Triglycerides reached 25% and 47%, respectively, the statistical significance was even lower than for the genus case. With the highest rank (class) the signal seems to disappear, suggesting that other genera of *Clostridia*, beyond *Clostridium*, while showing the beneficial correlation, probably do not participate in the Triglyceride lowering effect.

**Table 2:** Casual mediation analysis of the negative association of either Clostridium leptum species, Clostridium genus or Clostridia class with the blood Triglycerides or ApoB levels, as well as negative association of Roseburia genus with ApoB in context of the dietary intervention.

Mediator entity/target parameter	Statistic	Estimate	Lower CI	Upper CI	*p*-value
*C.leptum/TG*	ACME	−0.000182	−0.000544	0.00	0.252
	ADE	−0.001337	−0.002314	0.00	0.006
	Total Effect	−0.001519	−0.002476	0.00	<2*10^−16^
	Prop. Med.	0.114	−0.100	0.47	0.24
*Clostridium/TG*	ACME	−0.000232	−0.000589	0.00	0.136
	ADE	−0.001317	−0.002273	0.00	0.014
	Total Effect	−0.001550	−0.002496	0.00	<2*10^−16^
	Prop. Med.	0.146	−0.056	0.55	0.136
*Clostridia/TG*	ACME	−6.89e-05	−2.86e-04	0.00	0.43
	ADE	−1.43e-03	−2.41e-03	0.00	<2*10^−16^
	Total Effect	−1.50e-03	−2.46e-03	0.00	<2*10^−16^
	Prop. Med.	0.041	−0.100	0.25	0.526
*Clostridium/ApoB*	ACME	−0.0207	−0.0346	−0.01	<2*10^−16^
	ADE	−0.0288	−0.0639	0.01	0.11
	Total Effect	−0.0495	−0.0829	−0.02	0.01
	Prop. Med.	0.411	0.157	1.23	0.01
*Roseburia/ApoB*	ACME	0.000459	−0.004611	0.01	0.920
	ADE	−0.050068	−0.080881	−0.02	0.002
	Total Effect	−0.049608	−0.081674	−0.02	0.002
	Prop. Med.	−0.004	−0.177	0.09	0.918

*ACME* average causal mediation effects, *ADE* average direct effect; *Total Effect* the sum of direct and indirect effects, *Prop. Mediated* the proportion of the effect of Time on Triglyceride level mediated by the microbial entity, *Lower CI* lower 95% confidence interval, *Upper CI* upper 95% confidence interval.

Mediation analysis with the strongest genus-level correlation shown on the Fig. 6 (Roseburia—ApoB) has demonstrated a clearly zero mediation signal, setting the “no-signal” level for such analysis and making *Clostridium*—Triglyceride mediation result deserving some attention in comparison. Interestingly, mediation analysis of *Clostridium*—ApoB pair demonstrated very clear mediation signal of 41%, with pronounced statistical significance (Table 2). This signal disappears when we take the class-level taxa (*Clostridia*) into the analysis (not shown). This analysis suggests that *Clostridium* genus may participate in mediation of the diet-induced blood marker improvement, while other genera of the same class may be passengers rather than drivers of this process. Still, the purely statistical nature of this result does not allow to have an unequivocal conclusion about the functional link between *Clostridium* taxon abundance and the blood metabolic markers. The latter requires more detailed mechanistic studies that involve cultured microorganisms in question.

## Discussion

The study highlights the prevalence of the common effects of the dietary intervention with the three types of oils on the gut microbiota over specific effects observed in any given study group. The only exception is the magnitude and dynamics of the responses of the overall bacterial diversity that was more robust in the blended oil groups (especially Group C), as compared to the refined olive oil group. Previous research has shown similar effects of the three oil treatments on the blood lipid profiles suggesting an intervention-related boost in the overall content of unsaturated fatty acids as compared to the background diet of the participants rich in saturated fat, that is common to South East Asian diets^21^. Hence, the pronounced increase in intestinal *Clostridium leptum* species observed consistently in all the 3 groups of this study, may be related to the increase in the unsaturated fatty acids.

*C.leptum* is typically being discussed as a species contributing to butyrate production in the gut^31^, which mediates the beneficial influence of this species on a number of phenotypes, both related to intestinal health per se and beyond^32^. Another known mechanism of development of *C.leptum* beneficial effects on the hosts is via bile acid modification due to presence of 7-alpha-dehydroxylase, which is not found in *Bacteroides* species^33^.

There are several phenotypes that have a beneficial association with *C.leptum* abundance in the gut, according to previous studies. *C. leptum* was more abundant in lean women as compared to obese study participants^34^. It was found to be depleted in variety of health conditions, including those affecting body sites distant from the gut, such as non-small-cell lung cancer^35^.

With relevance to dietary patterns, *C.leptum* was depleted in a clinical study group that received a pro-inflammatory diet^36^, and increased with Goji berry supplementation, which helped to alleviate colitis in a mouse study^37^. Similar phenomena was also observed in a recent murine study; *C.leptum* was enriched following a dietary fiber supplementation across different fiber types^38^. It also increased in cecum of the piglets supplemented with inulin^39^. We mentioned earlier that the elevation of butyrate-producing capacity of the gut is the common theme for both dietary fiber and omega-3 PUFA supplementation. *C.leptum* is a known butyrate producer that can thrive in the gut microbial community in response to dietary oil supplementation, the phenomenon we see in the context of Singapore microbiome signatures. Reproducibility of this effect in other geographic locations and local microbiome composition contexts would require further studies.

While *Clostridiales* represent a consistent case of beneficial intestinal microbial taxa, this trend is not universal in other clades. Some taxons largely differ in the signs of their host health associations, which cannot be generalized towards the upper ranks of phylogeny. For example, with *Bacteriodaceae*, the situation is more complex because of a strong species-level dependence of the direction of the effect on the metabolic health. For example, the opposite role of different *Bacteroides* species in human metabolic health status was reported previously in an T2D study, where 5 *Bacteroides* species were found to be differentially abundant in T2D patients and controls^40^, including two (*B.plebeius* and *B.eggerthii*) enriched in T2D cases and three (*B.fragilis*, *B.uniformis* and *B.ovatus*) enriched in controls.

When associating microbiome features with clinical study factors or the blood markers of metabolic health, the choice of the most representative level of phylogeny rank is not obvious. Historically, the most used ranks used as descriptive of the microbiota changes are happened to be the two extreme ranks of the phylogeny: species and phylum (*Firmicutes* to *Bacteriodites* ratio belongs to the latter). We systematically scanned the intermediate ranks of the phylogeny for associations with both clinical trial factors and the blood markers of the metabolic health. At species level, *C leptum* was upregulated during the course of intervention but its correlation to plasma markers was relatively weak. At class level, *Clostridia* was both upregulated during the course of intervention and correlated with plasma markers across subjects. The involvement of *Clostridia* at the both ends of the diet-microbiota-health axis, i.e., both abundance-increase by the beneficial oils and the beneficial association with the blood markers of metabolic health, creates a stronger connection between the microbiota-level changes (this study) and the observed improvement of the plasma markers in the study participants in the course of the trial^21^. Still, our mediation analysis demonstrated that the stronger beneficial correlation with blood markers at the class level (*Clostridia*) does not translate to larger mediation signal (in fact, the mediation signal disappears at the class level). However, the genus level does show the mediation effect on the “diet–metabolic health” axis. Intermediate ranks of the same branch, between the genus and class, were shown to be beneficial for human health in other studies. For example, a recent large-scale study identified *Clostridiales* as the top health-beneficial taxon of the gut microbiota with widespread and statistically significant associations across multiple health state markers^41^. Notably, in that study employing serum lipid measures, the family *Clostridiacea* was associated not only with HDL but the largest-size HDL particles. In a recent extensive cross-sectional study that involved ~3400 US individuals and 150 phenotypic features, the order-level associations for *Clostridiales* were found to be both statistically most significant among other considered microbiome features and widespread across the study features, being consistently positively associated with health-related, and negatively—with disease-related features^42^.

Another aspect of the present study is to detect microbiota changes related to particular oil blends. While the multifactorial linear modeling could not detect reliable associations to the study groups, the paired tests for differences between the time points assessed separately in each group revealed a common pattern of increase in the magnitude and speed of the response from group A (refined olive oil) to groups B and C (blended oils), as seen in the changes in alpha-diversity over time. The oil compositional pattern that corresponds to this is concentration of Omega-3 PUFA (mainly ALA). If this projection is correct, then higher concentration of ALA/Omega3 PUFA promotes faster microbiota responses to the dietary intervention. Previous studies have demonstrated that Omega-3 dietary supplementation increase the abundance of the beneficial bacterial genera in the intestine^43^. Also, omega-3 PUFA consumption modulates obese intestinal microbiota by shifting it towards the state similar to normal weight subjects^44^. In mouse models, ALA was found to improve both the metabolic status and gut microbiota composition in animals fed with high-fat diet^45^. In a review focusing on the influence of dietary omega-3 on gut microbiota, multiple beneficial effects are listed, including stimulation of short-chain fatty acid production, beneficial effects on gut-brain axis and immunity^46^. In a rodent study, omega-3 supplementation induced intestinal expression of genes encoding fatty acid-oxidizing enzymes that contributing to a better metabolic phenotype^47^. Higher omega-3 to omega-6 ratio is able to attenuate the destructive effects of alcohol on intestinal homeostasis^48^. A large meta-analysis of omega-3 supplementation related clinical trials assessing cardiovascular health consequences^49^ summarized across 86 clinical trials (162,796 participants) came to conclusion that ALA supplementation can “slightly reduce risk of cardiovascular events and arrhythmia”. Since ALA is essential for humans and may be utilized as a precursor for synthesis of other omega-3 PUFA—EPA, DHA, which levels in the blood elevate following the ALA supplementation^50^—some effects of the ALA supply with the beneficial oils may be, in fact, mediated by EPA and/or DHA, suggesting to discuss the effect of omega-3 PUFA in general, before more mechanistic studies are conducted.

Alternative explanations of more robust effects of the oil blends compared to the olive oil may be based on phytonutrients that distinguish the blends from the olive oil (Supplementary Table 1). Oryzanol in the blended oils, from the rice bran oil, was previously shown^51^ to shift the microbial compositional pattern by lowering the *Firmicutes/Bacteroidetes* ratio, even though the change in the overall diversity was not reported. By supplying ferulic acid as one of its components, oryzanol increases the relative abundance of the ferulate-metabolizing bacteria such as *Lactobacilli*^52^, possibly shifting the gut microbial community equilibrium towards the healthier state by promoting short-chain fatty acids production and bile acids metabolism. Also, tocopherol content is higher in the oil blends compared to the refined olive oil, and its antimicrobial properties towards a number of pathogenic species in the gut are established^53^. Suppression of overrepresented pathogens by tocopherol may contribute to an increase in microbial diversity. On the other hand, higher content of phytosterols in the oil blends, as compared to olive oil, may increase the abundance of beneficial bacterial genera^54^, however, their effect on the diversity index per se has not been shown.

On a more mechanistic note, in a recent 6-week dietary intervention clinical trial, supplementation with omega-3 was found to increase isobutyrate and butyrate levels^55^. This may provide a connection between the enrichment in known butyrate-producing bacterial taxa we observed and omega-3-induced butyrate levels in an external study. The beneficial effect of butyrate at the microbiota-host interface and the key role of *Clostridia* in butyrate production are well established^56^. A combination of ecological and functional studies considering the intricate network of functional inter-dependencies between the intestinal bacterial species is required to gain detailed understanding of the phenomena observed in this study. The study provides the link between the beneficial effects of the new oil blends on the blood lipid profile and the microbiota changes in the gut, possibly via activation of the butyrate production.

## Methods

### Clinical intervention design

The 8-weeks single-blind, parallel-design, randomized controlled dietary intervention with 30 g per day of a dietary oil engaged 143 volunteers of Chinese origin. Selection criteria included borderline hypercholesterolemia (LDL cholesterol: 3.06–4.51 mmol/l), age in the range of 50 and 70 years and BMI ≤ 27.5. The study groups: Group A, refined olive oil; Group B, blended oil #1; Group C, blended oil #2. The oil blends contained refined rice bran oil, refined flaxseed oil, and refined sesame oil. The key differences between the oil blends in terms of fatty acid and phytonutrients content are given in Supplementary Table 1. Further details are given in the clinical report on this trial^21^. At 3 out of 5 time points of the original study—Week 0, Week 2, and Week 8—fecal samples were collected from participants who have chosen this option at the time of taking the informed consent, before the randomization was done. Hence, this metagenomics sub-study is restricted to 126 subjects (out of the 143 in the study, 88%) that had provided complete set of three fecal samples. Each intervention group had at least 40 participants (44, 42, and 40 in intervention groups A, B, and C, respectively, with gender composition of 21M/23F, 22M/20F, 18M/22F).

To test the degree of randomization, a post-randomization comparison of age, gender, weight, BMI, total body fat, blood pressure and levels of 10 serum markers (LDL, HDL, total cholesterol, triglycerides, ApoB, ApoA1, Total cholesterol to HDL ratio, ApoB to ApoA1 ratio, glucose and insulin) was performed. No statistically significant differences between the 3 intervention groups by any of those parameters were found^21^. We also tested for the baseline inter-group differences within the subset of 126 subjects who provided samples for metagenomic analysis. Similarly, no differences were detected in this case (Supplementary Table 2). The subjects’ adherence to the protocol was >98% for all three intervention groups, according to daily intervention food records.

The study was approved by a Domain Specific Review Board ethics committee, Singapore (reference: C/2018/00861). The participants signed the informed consent during their initial Screening and Consent visit.

The study is registered on clinicaltrials.gov (Identifier No. NCT03964857).

### Fecal samples collection and DNA isolation

Self-collection kits for the fecal samples were distributed to the study participants. Each kit contained a thermal bag, thermal pouches, ice packs (2 × 350 g), 50 ml Falcon tube for fresh (unfixed) sample and OmniGene Gut tube for DNA-preserved sample (DNA Genotek, Ottawa, Canada). The participants were instructed to collect the fecal samples within 24 h of a measurement visit at baseline (Week 0), then Week 2 and Week 8. The collected fecal samples were stored cold in the cooler bag with the provided pre-frozen ice packs.

Upon arrival, the fresh stool samples were aliquoted into 250 mg portions in 2 ml tubes and frozen at −80 °C. Samples fixed in OmniGene Gut were frozen at −80 °C upon arrival.

DNA isolation was performed using QIAamp PowerFecal spin column kit (Qiagen, USA). The cell disruption step was custom optimized for better balance between the DNA yield and integrity and consisted of four 1-min homogenization cycles at 6 m/s speed, with placing the tubes on ice for 15 s between the cycles and for 1 min at the end. All other steps were performed according to the manufacturer’s protocol. DNA integrity was checked with TapeStation 4200 (Agilent Technologies, USA) and purity was assessed by measuring A260/A280 and A260/A230 ratios with Qubit fluorometer (Thermo Fisher Scientific, USA).

### Metagenomic sequencing and low-level data processing

The purified DNA (1.5 mkg) was used for Illumina sequencing library construction (2x 150 bp, 300 bp insert). The library was sequenced on Illumina HiSeq2000 platform to the target depth of 3 Gbases per sample. This exceeds the depth used in the key publications from of the Human Microbiome Project (for example, 2 Gbases per sample in IBD study^57^) and is chosen to ensure the above-conventional level of the sensitivity of the species detection.

After adaptor sequences removal, the FASTQ files were subject to a 2-stage filtering: (1) with *Trimmomatic*^58^ version 0.33 tuned to retain the reads that pass a minimal average Q-score of 28 in a sliding window of size 3 and remained at least 50 bp long after the procedure and (2) with *kneaddata*^59^ version 0.6.1 tool to remove the reads matching either host (*H. sapiens* hg19) genome or rRNA gene sequences of various origin from Silva^60^ database. Pairs of reads with both forward and reverse read passed the filtering criteria were kept for all the subsequent analyses.

The pre-processed sequencing reads were analyzed with *Metaphlan version 2*^61^ in pair-end mode. The resulting abundance table was normalized to get the sum of abundances of all the species in each sample equal to 1. Over 99% of reads were mapped to *Bacteria* (Supplementary Table 3), and the downstream results are interpreted in terms of bacterial phylogeny.

Pathway-centric representation of the data was generated with HUMAnN2^62^.

### Downstream data analysis

Data analysis was performed in an R statistical environment^63^ using a combination of custom scripting with *vegan*, *microbiome, gplots, Maaslin2* and *ggplot2* packages.

### Multivariate analysis and differential abundance testing

With the strong contribution of subject-specific microbiome composition to the overall data structure (see Results), direct comparison of data between the groups is prone to false positives, and indeed, in preliminary tests, we noticed that the differential abundance signatures detected this way are overwhelmed by the outlier species-driven signals and cannot be interpreted as the effects of the dietary intervention: some apparent “study group effects” were visible at Week 0, before the start of the actual clinical trial (not shown). This phenomenon may drive false-positive results in some published studies that use direct comparison of the data between the study groups. To overcome this issue, we leveraged the existence of a time course in our study and used two complementary statistical approaches (below).

Differential abundance of microbiota features relevant to the clinical trial (temporal changes and differences between the study groups) were assessed in multivariate analysis setting using *Maaslin2*^64^ version 1.3.2. The analysis was run with *min_abundance* = 0.0001 and *min_prevalence* = 0.1 parameters, setting parameters of interest (Group and Time) as target variables, and subject ID as random effect. The latter (achieved by *random_effects* = “*Subject*”parameter) switched Maaslin2 analysis to Linear Mixed-Effects Models mode that leverages the longitudinal design of the study and suppresses potential false-positive results coming from extensive differences in microbial composition of the individual intestines (see Results).

As an alternative, paired two-sided Mann–Whitney–Wilcoxon test between different time points was applied to detect dietary intervention-related changes in the microbiota features. To obtain the false-discovery rates (FDR), the *p*-values of the Wilcoxon test were corrected according to Benjamini–Hochberg procedure^65^.

Associations between the microbial taxa abundance and the blood markers of metabolic health were performed using *Maaslin2* by correlating the levels of a blood marker with the relative taxa abundance in the corresponding fecal samples, using the Linear Mixed-Effects model described above, across the dataset of the 378 subject—time point combinations.

Causal Mediation Analysis^30^ was performed using *mediation* R package and was based on the two linear models: (1) abundance of the bacterial taxa as a function of the intervention dynamics, corrected for the Subject ID and (2) level of the blood marker as a function of the bacterial taxa, intervention dynamics and the Subject ID. Subsequently, the models were used as arguments for the “mediate” function, with “mediator” parameter set to the bacterial taxa and “treatment”—to the day of intervention.

PERMANOVA was performed using *vegan* R package. Total variance explained by each variable was assessed by marginal effects and their corresponding statistical significance reported accordingly. For variables of repeated measurements such as group and time, the variances and statistical significances were computed by a permutation test that was designed around blocks of samples repeated across time points. For inter-individual variances, variances and statistical significances were computed by a permutation that was freely randomized.

### Reporting summary

Further information on research design is available in the Nature Research Reporting Summary linked to this article.

## Supplementary information

Reporting Summary

Supp Table 2

Supp Table 5

Supp Table 6

Supp Table 7

Supp Table 8

Supp Table 9

Supp Table 10

New SI requested by ME

Supp Table 3

Supp Table 11

## Data Availability

The data that support the findings of this study are openly available in NCBI Sequence Read Archive, BioProject PRJNA728374. The clinical metadata part of this dataset, utilized here for association analysis, was first discussed in the initial publication^21^.

## References

[1] Dong TS, Gupta A (2019). Influence of early life, diet, and the environment on the microbiome. Clin. Gastroenterol. Hepatol..

[2] Zmora, N., Soffer, E. & Elinav, E. Transforming medicine with the microbiome. *Sci. Transl. Med.* **11**, 10.1126/scitranslmed.aaw1815 (2019). 10.1126/scitranslmed.aaw1815 30700573

[3] Sanchez-Tapia M, Tovar AR, Torres N (2019). Diet as regulator of gut microbiota and its role in health and disease. Arch. Med. Res..

[4] Zegarra-Ruiz DF (2019). A diet-sensitive commensal Lactobacillus strain mediates TLR7-dependent systemic autoimmunity. Cell Host Microbe.

[5] Muralitharan RR (2020). Microbial peer pressure: the role of the gut microbiota in hypertension and its complications. Hypertension.

[6] Singer-Englar T, Barlow G, Mathur R (2019). Obesity, diabetes, and the gut microbiome: an updated review. Expert Rev. Gastroenterol. Hepatol..

[7] Molendijk, I., van der Marel, S. & Maljaars, P. W. J. Towards a food pharmacy: immunologic modulation through diet. *Nutrients* **11**, 10.3390/nu11061239 (2019). 10.3390/nu11061239 PMC6627620 31159179

[8] Johnson SS (2019). Editor’s desk: masterful microbes: the gut microbiome and food as medicine. Am. J. Health Promot.

[9] Leshem, A., Segal, E. & Elinav, E. The gut microbiome and individual-specific responses to diet. *mSystems* **5**, 10.1128/mSystems.00665-20 (2020). 10.1128/mSystems.00665-20 PMC7527138 32994289

[10] Integrative, H. M. P. R. N. C. (2019). The integrative human microbiome project. Nature.

[11] Wilson AS (2020). Diet and the human gut microbiome: an international review. Dig. Dis. Sci..

[12] Makki K, Deehan EC, Walter J, Backhed F (2018). The impact of dietary fiber on gut microbiota in host health and disease. Cell Host Microbe.

[13] Barkas, F., Nomikos, T., Liberopoulos, E. & Panagiotakos, D. Diet and cardiovascular disease risk among individuals with familial hypercholesterolemia: systematic review and meta-analysis. *Nutrients* **12**10.3390/nu12082436 (2020). 10.3390/nu12082436 PMC7468930 32823643

[14] Albracht-Schulte K (2018). Omega-3 fatty acids in obesity and metabolic syndrome: a mechanistic update. J. Nutritional Biochem..

[15] Schoeler M, Caesar R (2019). Dietary lipids, gut microbiota and lipid metabolism. Rev. Endocr. Metab. Disord..

[16] Sonnenburg JL, Backhed F (2016). Diet-microbiota interactions as moderators of human metabolism. Nature.

[17] Thomas SS, Cha YS, Kim KA (2020). Effect of vegetable oils with different fatty acid composition on high-fat diet-induced obesity and colon inflammation. Nutr. Res. Pr..

[18] Nakaya K, Ikewaki K (2018). Microbiota and HDL metabolism. Curr. Opin. Lipidol..

[19] Wisniewski, P. J., Dowden, R. A. & Campbell, S. C. Role of dietary lipids in modulating inflammation through the gut microbiota. *Nutrients* **11**, 10.3390/nu11010117 (2019). 10.3390/nu11010117 PMC6357048 30626117

[20] Wan XZ (2019). Anti-diabetic activity of PUFAs-rich extracts of Chlorella pyrenoidosa and Spirulina platensis in rats. Food Chem. Toxicol..

[21] Haldar S (2020). Two blends of refined rice bran, flaxseed, and sesame seed oils affect the blood lipid profile of chinese adults with borderline hypercholesterolemia to a similar extent as refined olive oil. J. Nutr..

[22] Tamanai-Shacoori Z (2017). Roseburia spp.: a marker of health?. Future Microbiol..

[23] Scheiman J (2019). Meta-omics analysis of elite athletes identifies a performance-enhancing microbe that functions via lactate metabolism. Nat. Med..

[24] Hjorth MF (2019). Prevotella-to-Bacteroides ratio predicts body weight and fat loss success on 24-week diets varying in macronutrient composition and dietary fiber: results from a post-hoc analysis. Int. J. Obes..

[25] Wang C (2020). The genus Sutterella is a potential contributor to glucose metabolism improvement after Roux-en-Y gastric bypass surgery in T2D. Diabetes Res. Clin. Pr..

[26] Fu J (2015). The gut microbiome contributes to a substantial proportion of the variation in blood lipids. Circulation Res..

[27] Wang Z, Koonen D, Hofker M, Fu J (2016). Gut microbiome and lipid metabolism: from associations to mechanisms. Curr. Opin. Lipidol..

[28] Matey-Hernandez ML (2018). Genetic and microbiome influence on lipid metabolism and dyslipidemia. Physiol. Genomics.

[29] Zeng MY, Inohara N, Nunez G (2017). Mechanisms of inflammation-driven bacterial dysbiosis in the gut. Mucosal Immunol..

[30] Imai K, Keele L, Tingley D (2010). A general approach to causal mediation analysis. Psychol. Methods.

[31] Louis P, Flint HJ (2009). Diversity, metabolism and microbial ecology of butyrate-producing bacteria from the human large intestine. FEMS Microbiol. Lett..

[32] Canani RB (2011). Potential beneficial effects of butyrate in intestinal and extraintestinal diseases. World J. Gastroenterol.: WJG.

[33] Stellwag EJ, Hylemon PB (1978). Characterization of 7-alpha-dehydroxylase in Clostridium leptum. Am. J. Clin. Nutr..

[34] Teixeira TFS (2013). Faecal levels of Bifidobacterium and Clostridium coccoides but not plasma lipopolysaccharide are inversely related to insulin and HOMA index in women. Clin. Nutr..

[35] Gui Q (2020). The association between gut butyrate-producing bacteria and non-small-cell lung cancer. J. Clin. Lab Anal..

[36] Zheng J (2020). Dietary inflammatory potential in relation to the gut microbiome: results from a cross-sectional study. Br. J. Nutr..

[37] Kang Y, Yang G, Zhang S, Ross CF, Zhu MJ (2018). Goji berry modulates gut microbiota and alleviates colitis in il-10-deficient mice. Mol. Nutr. Food Res..

[38] Raza GS (2019). Hypocholesterolemic effect of the lignin-rich insoluble residue of brewer’s spent grain in mice fed a high-fat diet. J. Agric. Food Chem..

[39] Passlack N, Vahjen W, Zentek J (2015). Dietary inulin affects the intestinal microbiota in sows and their suckling piglets. BMC Vet. Res..

[40] Medina-Vera I (2019). A dietary intervention with functional foods reduces metabolic endotoxaemia and attenuates biochemical abnormalities by modifying faecal microbiota in people with type 2 diabetes. Diabetes Metab..

[41] Vojinovic D (2019). Relationship between gut microbiota and circulating metabolites in population-based cohorts. Nat. Commun..

[42] Manor O (2020). Health and disease markers correlate with gut microbiome composition across thousands of people. Nat. Commun..

[43] Watson H (2018). A randomised trial of the effect of omega-3 polyunsaturated fatty acid supplements on the human intestinal microbiota. Gut.

[44] Coelho OGL, Candido FG, Alfenas RCG (2019). Dietary fat and gut microbiota: mechanisms involved in obesity control. Crit. Rev. Food Sci. Nutr..

[45] Gao, X. et al. Correlations between alpha-linolenic acid-improved multitissue homeostasis and gut microbiota in mice fed a high-fat diet. *mSystems* **5**, 10.1128/mSystems.00391-20 (2020). 10.1128/mSystems.00391-20 PMC7646523 33144308

[46] Costantini, L., Molinari, R., Farinon, B. & Merendino, N. Impact of omega-3 fatty acids on the gut microbiota. *Int. J. Mol. Sci.* **18**, 10.3390/ijms18122645 (2017). 10.3390/ijms18122645 PMC5751248 29215589

[47] Kroupova, P. et al. Omega-3 phospholipids from krill oil enhance intestinal fatty acid oxidation more effectively than omega-3 triacylglycerols in high-fat diet-fed obese mice. *Nutrients* **12**, 10.3390/nu12072037 (2020). 10.3390/nu12072037 PMC7400938 32660007

[48] Warner DR (2019). Decreased omega-6:omega-3 PUFA ratio attenuates ethanol-induced alterations in intestinal homeostasis, microbiota, and liver injury. J. Lipid Res..

[49] Abdelhamid AS (2020). Omega-3 fatty acids for the primary and secondary prevention of cardiovascular disease. Cochrane Database Syst. Rev..

[50] Brenna JT (2009). alpha-Linolenic acid supplementation and conversion to n-3 long-chain polyunsaturated fatty acids in humans. Prostaglandins Leukot. Ess. Fat. Acids.

[51] Kozuka C (2017). Marked augmentation of PLGA nanoparticle-induced metabolically beneficial impact of gamma-oryzanol on fuel dyshomeostasis in genetically obese-diabetic ob/ob mice. Drug Deliv..

[52] Tamura M, Hori S, Hoshi C, Nakagawa H (2012). Effects of rice bran oil on the intestinal microbiota and metabolism of isoflavones in adult mice. Int. J. Mol. Sci..

[53] Hartmann, M. S., Mousavi, S., Bereswill, S. & Heimesaat, M. M. Vitamin E as promising adjunct treatment option in the combat of infectious diseases caused by bacterial including multi-drug resistant pathogens-Results from a comprehensive literature survey. *Eur*. *J. Microbiol. Immunol. (Bp)*, 10.1556/1886.2020.00020 (2020). 10.1556/1886.2020.00020 PMC7753978 33151163

[54] Cuevas-Tena M (2018). Plant sterols and human gut microbiota relationship: an in vitro colonic fermentation study. J. Funct. Foods.

[55] Vijay A, Astbury S, Le Roy C, Spector TD, Valdes AM (2021). The prebiotic effects of omega-3 fatty acid supplementation: A six-week randomised intervention trial. Gut Microbes.

[56] Furusawa Y (2013). Commensal microbe-derived butyrate induces the differentiation of colonic regulatory T cells. Nature.

[57] Lloyd-Price J (2019). Multi-omics of the gut microbial ecosystem in inflammatory bowel diseases. Nature.

[58] Bolger AM, Lohse M, Usadel B (2014). Trimmomatic: a flexible trimmer for Illumina sequence data. Bioinformatics.

[59] Huttenhower. https://huttenhower.sph.harvard.edu/kneaddata/. (2019).

[60] Quast C (2013). The SILVA ribosomal RNA gene database project: improved data processing and web-based tools. Nucleic Acids Res..

[61] Segata N (2012). Metagenomic microbial community profiling using unique clade-specific marker genes. Nat. Methods.

[62] Franzosa EA (2018). Species-level functional profiling of metagenomes and metatranscriptomes. Nat. Methods.

[63] Team, R. C. R. *A Language and Environment for Statistical Computing* (Team, R. C. R, 2015).

[64] Morgan XC (2012). Dysfunction of the intestinal microbiome in inflammatory bowel disease and treatment. Genome Biol..

[65] Benjamini Y, Hochberg Y (1995). Controlling the false discovery rate: a practical and powerful approach to multiple testing. J. R. Stat. Soc. Ser. B.

