# Supplementary material for: Gut microbiome responses to dietary intervention with hypocholesterolemic vegetable oils
Source: NPJ Biofilms Microbiomes. 2022 Apr 11;8:24. doi: 10.1038/s41522-022-00287-y (PMC9001705; doi:10.1038/s41522-022-00287-y)
Supplement: Supplementary file 1 — Reporting Summary [file 41522_2022_287_MOESM1_ESM.pdf]

nature portfolio

Corresponding author(s): Oleg Moskvin

Last updated by author(s): 01/27/2022

Reporting Summary

Nature Portfolio wishes to improve the reproducibility of the work that we publish. This form provides structure for consistency and transparency in reporting. For further information on Nature Portfolio policies, see our Editorial Policies and the Editorial Policy Checklist.

Statistics

For all statistical analyses, confirm that the following items are present in the figure legend, table legend, main text, or Methods section.

n/a	Confirmed	
☐	☒	The exact sample size (*n*) for each experimental group/condition, given as a discrete number and unit of measurement
☐	☒	A statement on whether measurements were taken from distinct samples or whether the same sample was measured repeatedly
☐	☒	The statistical test(s) used AND whether they are one- or two-sided<br>*Only common tests should be described solely by name; describe more complex techniques in the Methods section.*
☐	☒	A description of all covariates tested
☐	☒	A description of any assumptions or corrections, such as tests of normality and adjustment for multiple comparisons
☐	☒	A full description of the statistical parameters including central tendency (e.g. means) or other basic estimates (e.g. regression coefficient) AND variation (e.g. standard deviation) or associated estimates of uncertainty (e.g. confidence intervals)
☐	☒	For null hypothesis testing, the test statistic (e.g. *F*, *t*, *r*) with confidence intervals, effect sizes, degrees of freedom and *P* value noted<br>*Give P values as exact values whenever suitable.*
☒	☐	For Bayesian analysis, information on the choice of priors and Markov chain Monte Carlo settings
☒	☐	For hierarchical and complex designs, identification of the appropriate level for tests and full reporting of outcomes
☐	☒	Estimates of effect sizes (e.g. Cohen's *d*, Pearson's *r*), indicating how they were calculated

Our web collection on statistics for biologists contains articles on many of the points above.

Software and code

Policy information about availability of computer code

Data collection	No data collection software was used
Data analysis	As stated in the Methods, a combination of freely available software packages was used. Sufficient details were given to reproduce the results with the repository-posted complete datasets, from software version numbers to particular values of the parameters supplied to functions.

For manuscripts utilizing custom algorithms or software that are central to the research but not yet described in published literature, software must be made available to editors and reviewers. We strongly encourage code deposition in a community repository (e.g. GitHub). See the Nature Portfolio guidelines for submitting code & software for further information.

Data

Policy information about availability of data

All manuscripts must include a data availability statement. This statement should provide the following information, where applicable:

- Accession codes, unique identifiers, or web links for publicly available datasets
- A description of any restrictions on data availability
- For clinical datasets or third party data, please ensure that the statement adheres to our policy

The data that support the findings of this study are openly available in NCBI Sequence Read Archive, BioProject PRJNA728374.

Field-specific reporting

Please select the one below that is the best fit for your research. If you are not sure, read the appropriate sections before making your selection.

☒ Life sciences ☐ Behavioural & social sciences ☐ Ecological, evolutionary & environmental sciences

For a reference copy of the document with all sections, see nature.com/documents/nr-reporting-summary-flat.pdf

Life sciences study design

All studies must disclose on these points even when the disclosure is negative.

Sample size	Sample size estimation was performed for the original study focused on clinical outcomes (Haldar, 2020). Briefly, GLIMMPSE software (http://glimmpse.SampleSizeShop.org/) was used in determining the sample size. The estimated maximum sample size was 34 volunteers per treatment and a total sample size of 102. The actual number of samples exceeded this estimate.
Data exclusions	The main subject exclusion criteria were BMI (kg/m2) >27.5; smoking; following any special diets (for medical or aesthetic reasons); having a medical history of heart, liver, kidney, blood, or thyroid dysfunction; having diabetes or acute gastrointestinal disorders; having any major gastrointestinal surgery; and for women, being pre- or perimenopausal. For the metagenomic data, a subjects with incomplete set of 3 time points were excluded (still leaving the numbers of subjects per group - 44, 42 and 40 in intervention groups A, B and C - that exceed the sample size estimate.
Replication	Special care was taken to study the metagenomic data structure and assessing relative influence of the study parameters on data variance. Analytical methods were critically assessed in the light of the requirement to avoid false positive calls driven by individual microbiota compositions of the subjects. Throughout the analysis, strict control for those differences was applied via Linear Mixed-Effects Models, producing the most conservative result, with the selected microbial features readily relatable to the existing knowledge in the area.
Randomization	Volunteers were randomly assigned to the intervention arms using GraphPad prism, separately for males and females. To test the degree of randomization, a post-randomization comparison of age, gender, weight, BMI, total body fat, blood pressure and levels of 10 serum markers (LDL, HDL, total cholesterol, triglycerides, ApoB, ApoA1, Total cholesterol to HDL ratio, ApoB to ApoA1 ratio, glucose and insulin) was performed. No statistically significant differences between the 3 intervention groups by any of those parameters were found.
Blinding	The sample collection, DNA isolation, sequencing, data QC and quality filtering, translation of the sequencing data into microbial abundance, combining the data into the master table, final abundance filtering and normalization were done before the group assignment of the samples were revealed for the association studies.

Reporting for specific materials, systems and methods

We require information from authors about some types of materials, experimental systems and methods used in many studies. Here, indicate whether each material, system or method listed is relevant to your study. If you are not sure if a list item applies to your research, read the appropriate section before selecting a response.

Materials & experimental systems

n/a	Involved in the study	
☒	☐	Antibodies
☒	☐	Eukaryotic cell lines
☒	☐	Palaeontology and archaeology
☒	☐	Animals and other organisms
☐	☒	Human research participants
☐	☒	Clinical data
☒	☐	Dual use research of concern

Methods

n/a	Involved in the study	
☒	☐	ChIP-seq
☒	☐	Flow cytometry
☒	☐	MRI-based neuroimaging

Human research participants

Policy information about studies involving human research participants

Population characteristics	The study group consisted of borderline hypercholesterolemic men and women of Chinese ethnic origin, 50–70 y of age. Borderline hypercholesterolemia was defined as having measured serum LDL cholesterol between 3.06 and 4.51 mmol/L, based on ±10% of the Adult Treatment Panel III (ATP III) classification.
Recruitment	The volunteers who took part in this study were recruited via advertisements in local newspapers, in community groups, approaching potential volunteers from ClinicalNutrition Research Center (CNRC), Singapore, recruitment database, as well as through word of mouth. Relatives of the researchers involved in the study were not eligible to participate.
Ethics oversight	The study was approved by a Domain Specific Review Board ethics committee, Singapore (reference: C/2018/00861).

Note that full information on the approval of the study protocol must also be provided in the manuscript.

Clinical data

Policy information about clinical studies

All manuscripts should comply with the ICMJE guidelines for publication of clinical research and a completed CONSORT checklist must be included with all submissions.

Clinical trial registration	The study is registered on clinicaltrials.gov (Identifier No. NCT03964857).
Study protocol	https://clinicaltrials.gov/ct2/show/NCT03964857 Also, doi: https://doi.org/10.1093/jn/nxaa274
Data collection	The trial was conducted between November 2018 and May 2019. Participants delivered their fecal samples to ClinicalNutrition Research Center (CNRC), Singapore.
Outcomes	All the details are given in https://clinicaltrials.gov/ct2/show/NCT03964857 and doi: https://doi.org/10.1093/jn/nxaa274
