# Supplementary material for: Gut microbiome responses to dietary intervention with hypocholesterolemic vegetable oils
Source: NPJ Biofilms Microbiomes. 2022 Apr 11;8:24. doi: 10.1038/s41522-022-00287-y (PMC9001705; doi:10.1038/s41522-022-00287-y)
Supplement: Supplementary file 9 — New SI requested by ME [file 41522_2022_287_MOESM9_ESM.pdf]

Supplementary Table 1.
A subset of the compositional information on the dietary intervention oils (grams per 100g of total fatty acids). **a**, olive oil; **b**, Blend 1; **c**, Blend 2.

Class of fatty acids	Units	a	b	c
**Saturated**	g/100g total fatty acids	16.6	14.9	13.6
**Monounsaturated**		70.8	32.9	28.1
**n-6 Polyunsaturated**		11.5	30.1	23.1
**n-3 Polyunsaturated**		1.0	21.4	34.5
**ALA**		0.8	20.9	30.4
**n-3/n-6 PUFA**	ratio	0.09	0.70	1.44
**phytosterol**	mg/kg oil	1780	7600	5700
**tocopherol**		186	325	315
**oryzanol**		NA	300	125

Supplementary Table 2.
Summaries of 13 numeric parameters related to the 126 metagenomic study subjects within each study group. Columns B-G, mean (B, D, F) and standard deviation (C, E, G) values. Columns H-M, p-values of Wilcoxon test for the 3 pairwise inter-group comparisons before (H, J, L) and after (I, K, M) correction for multiple testing using Benjamini-Hochberg procedure. Available as Supplementary_Table_2.xlsx

Supplementary Table 3.
Per-sample assignments of the mapped sequencing reads to the kingdoms of life, percentage. BOBXXX, an internal subject ID of the study; “1”, “2” and “3” suffixes, the time point of the sample collection (Week 0, Week 2 and Week 8, respectively). Available as Supplementary_Table_3.xlsx

Supplementary Table 4. PERMANOVA analysis of contributions of the study factors to the variance of microbial abundance data.

Factors	ID	Df	SumsOfSq	R2	F	Pr(>F)
**Time / Group**	Group	2	2.79	0.00753	1.4225	0.000999
	Time	2	2.1	0.00568	1.0734	0.000999
	Residuals	373	365.36	0.98679		
	Total	377	370.25	1		
**Subject**	Subject	125	178.34	0.48167	1.8734	0.000999
	Residuals	252	191.91	0.51833		
	Total	377	370.25	1		

Supplementary Table 5.
Abundance of 6 ranks of phylogeny (species, genus, family, order, class, phylum) in the entire BOB dataset and at the per-sample level. Column B, share in the entire pooled dataset of 378 samples; column C, lowest share in a sample; column D, highest share in a sample; column E, share of the samples (out of 378) with non-zero detection of the current entity; column F, share of subjects (out of 126) with non-zero detection of the entity at at least 1 out of 3 time points. Available as Supplementary_Table_5.xlsx

Supplementary Table 6.
Associations between species abundance and the clinical trial factors from MaAsLin2 multivariate model. Available as Supplementary_Table_6.xlsx

Supplementary Table 7.
Associations between species abundance and the clinical trial factors from paired test for the difference between the time points. Column A, species name; Column B, signed fold change; Column C, absolute difference (in scaled-to-1 units) between the mean values for the 2 timepoints. Available as Supplementary_Table_7.xlsx

Supplementary Table 8. Associations between abundance of 5 ranks of phylogeny beyond the species (genus, family, order, class, phylum) and the clinical trial factors from *MaAsLin2* multivariate model. Available as Supplementary_Table_8.xlsx

Supplementary Table 9.
Associations of the taxa abundance at various phylogeny ranks with the blood markers of the metabolic health. Values of –log10(FDR) signed by the direction of the association are shown. Available as Supplementary_Table_9.xlsx

Supplementary Table 10.
Associations between pathways abundance and the clinical trial factors from MaAsLin2 multivariate model. Available as Supplementary_Table_10.xlsx

Supplementary Table 11.
Associations between pathways abundance and the clinical trial factors from paired test for the difference between the time points. Columns' designations are identical to those of the Supplementary Table 6. Available as Supplementary_Table_11.xlsx
